# Within-subject variability in human retinal nerve fiber bundle width

**DOI:** 10.1371/journal.pone.0223350

**Published:** 2019-10-16

**Authors:** William H. Swanson, Brett J. King, Stephen A. Burns

**Affiliations:** School of Optometry, Indiana University, Bloomington, Indiana, United States of America; Doheny Eye Institute/UCLA, UNITED STATES

## Abstract

With the growing availability of high-resolution imaging there has been increased interest in developing new metrics for integrity of the retinal nerve fiber layer. In particular, it has been suggested that measurement of width of retinal nerve fiber bundles (RNFBs) may be useful in glaucoma, due to low between-subject variability in mean RNFB width. However, there have also been reports of substantial within-subject variability in the width of individual RNFBs. To assess within-subject variability as a potential source of selection bias in measurements of RNFB width, we used an adaptive optics scanning laser ophthalmoscope (AOSLO) to measure widths of individual RNFBs in one eye each of 11 young adults in good ocular health. In a pilot study we analyzed a large AOSLO image of RNFL in one participant then, based on those findings, in the main study we used AOSLO to image a smaller region in 10 additional healthy young adults. The pilot study of one eye found RNFB widths ranging from 10 μm to 44 μm. This suggested that biological variability was too high for measuring small changes arising from disease processes. This was confirmed in measurements of 10 eyes in the main study, RNFB widths ranged from 9 μm to 55 μm and every eye had large within-subject variability (exceeding 19 μm in all eyes) in RNFB width for nearby bundles. The within-subject variability in RNFB width, as well as variation in the width of single RNFBs over relatively short distances (<300 um) depending on the precise location of measurement, suggests that bundle width measurements would be highly susceptible to selection bias and therefore of limited clinical use.

## Introduction

Over the past two decades, the ability of clinical retinal imaging to quantify thickness of the circumpapillary retinal nerve fiber layer (RNFL) has changed how glaucoma is managed. [1] The axial resolution of the original optical coherence tomography (OCT) systems was high enough to estimate RNFL thickness through image segmentation, but the lateral resolution was too low to identify individual retinal nerve fiber bundles (RNFBs). Therefore, RNFL thickness became the standard clinical measure in managing patients with glaucoma. Spectral domain (SD) OCT devices can image much larger areas with higher resolution, making it possible to identify individual RNFBs where they are relatively sparse, such as the temporal raphe. [2, 3] In research labs, the increased lateral resolution of Adaptive Optics (AO) systems has made it possible to identify individual RNFBs across the retina. [4–7]

With the growing availability of high-resolution imaging there has been increased interest in developing new methods for assessing integrity of the retinal nerve fiber layer. [8–15] It is increasingly evident that a fundamental limit to the ability to assess glaucomatous damage in an individual patient is the between-subject variability among people in good ocular health [16–22]. There is substantial normal between-subject variability in circumpapillary RNFL thickness [19] and in shape of 2-dimensional thickness maps. [20] In comparison, a PLOS ONE paper from the Takayama lab reported that there is low between-subject variability in mean RNFB width, [6] and the same lab suggested that measurement of RNFB width may be useful in glaucoma. [23] However, there is also a report of substantial within-subject variability in width of individual RNFBs. [4]

It is common to focus on between-subject variability, because this is used to determine the normal range (e.g., 5^th^ & 1^st^ percentiles for RNFL thickness). However, for methods which require an individual to choose locations for measurements, within-subject variability is important as a potential source of bias. The Takayama lab estimated bundle width as an average of widths measured at 9–24 locations chosen by each of two graders. If within-subject variability in bundle width is low, choices of locations should have little impact on the mean widths. However, if within-subject variability in bundle width is high then an unconscious bias in choices of locations could have a substantial impact. If within-subject variability in bundle width is indeed as large as the Miller lab indicated, then it would be possible for unconscious selection bias to lead graders to select locations where bundle width was thinner than the average in eyes of patients with glaucoma, and locations where bundle width was thicker than average in healthy eyes. Such selection bias could then cause the appearance that bundle width was thinner in eyes with glaucoma. Similarly, difference between laboratories in how they selected bundles to measure could lead to irreproducible results. To assess within-subject variability as a potential source of bias in measurements of RNFB width, we used an adaptive optics scanning laser ophthalmoscope (AOSLO) to measure widths of individual RNFBs in one eye each of 11 young adults in good ocular health.

## Methods

### Participants

One healthy young adult had previously been imaged, [5] and this montage was used in the pilot study. Then, 10 additional healthy young adults ages 23–33 years (median 25 years) were recruited. Seven were females and 3 were males. Using the standard National Institutes of Health categories, 7 self-identified as non-Hispanic White, 2 as non-Hispanic Asian, and 1 as Hispanic African-American. All subjects were considered free of eye disease after a recent comprehensive ophthalmic examination: normal optic cup-disc ratio, open anterior chamber angles, spherical equivalent refractive error within -6 D to +3 D, best- corrected visual acuity of 20/20 or better. Subjects with a history of ocular disease or eye surgery were excluded. The research for this study adhered to the tenets of the Declaration of Helsinki and was approved by the Institutional Review Board at Indiana University. Informed consent was obtained from each participant after explanation of the procedures and goals of the study, before testing began.

### Equipment

The custom Indiana AOSLO system has been described previously. [24] In brief, the current AOSLO uses a supercontinuum light source for both wavefront sensing and retinal imaging. Wavefront sensing and imaging is performed at 775 nm and a second optical channel performs simultaneous retinal imaging at 830 nm. The total light level is safe according to American National Standards Institute standards (ANSI Z136). The system uses two deformable mirrors. One mirror with 52 actuators is used to correct low-order aberrations and one with 144 actuators is used to correct high-order aberrations, and the two mirrors operate in a woofer-tweeter configuration. [25] Two scanners are used to create the raster on the retina with a frame rate of approximately 30 frames per second. The lateral resolution of the system is approximately 2 μm with an 8-mm pupil. The imaging field could be steered across the retina through a 30° angle. [5]

An IOLMaster (v5, Carl Zeiss Meditec) was used to measure axial length and corneal curvature for both eyes of each participant.

### Imaging protocols

The protocol for imaging used in the pilot study has been described in the paper where much of this montage was previously published. [5] For the protocol in the main study, each of the 10 participants had their right eye dilated using 1% tropicamide and 2.5% phenylephrine before images were taken. The area to be imaged was 8° vertical by 15° horizontal. A confocal aperture was used and the scan was set to 2.0° wide by 1.8° tall, with ~1 micron per pixel. For each eye there were numerous scans (from 83 to 134 scans, median 123 scans) beginning between the fovea and the disc and extending to the oblique region of the temporal raphe. [26]

The AOSLO images were processed and montaged using customized software (written in MatLab; MathWorks, Natick, MA, USA).

### Measuring RNFB widths

A single trained staff member measured all RNFB widths using Adobe Photoshop CC (Adobe Systems, Inc., San Jose, CA, USA). Using the line tool, a line was drawn perpendicular to the edges of the RNFB and Photoshop returned the length of the line in microns. The measurement scale in Photoshop was set so that 1 pixel equaled 1 micron. After measurements were complete, the method of Bennett [27] was used to adjust scaling for axial length; this led to changes in RNFB width from -5% to +3%.

### Pilot study

**Fig 1:**
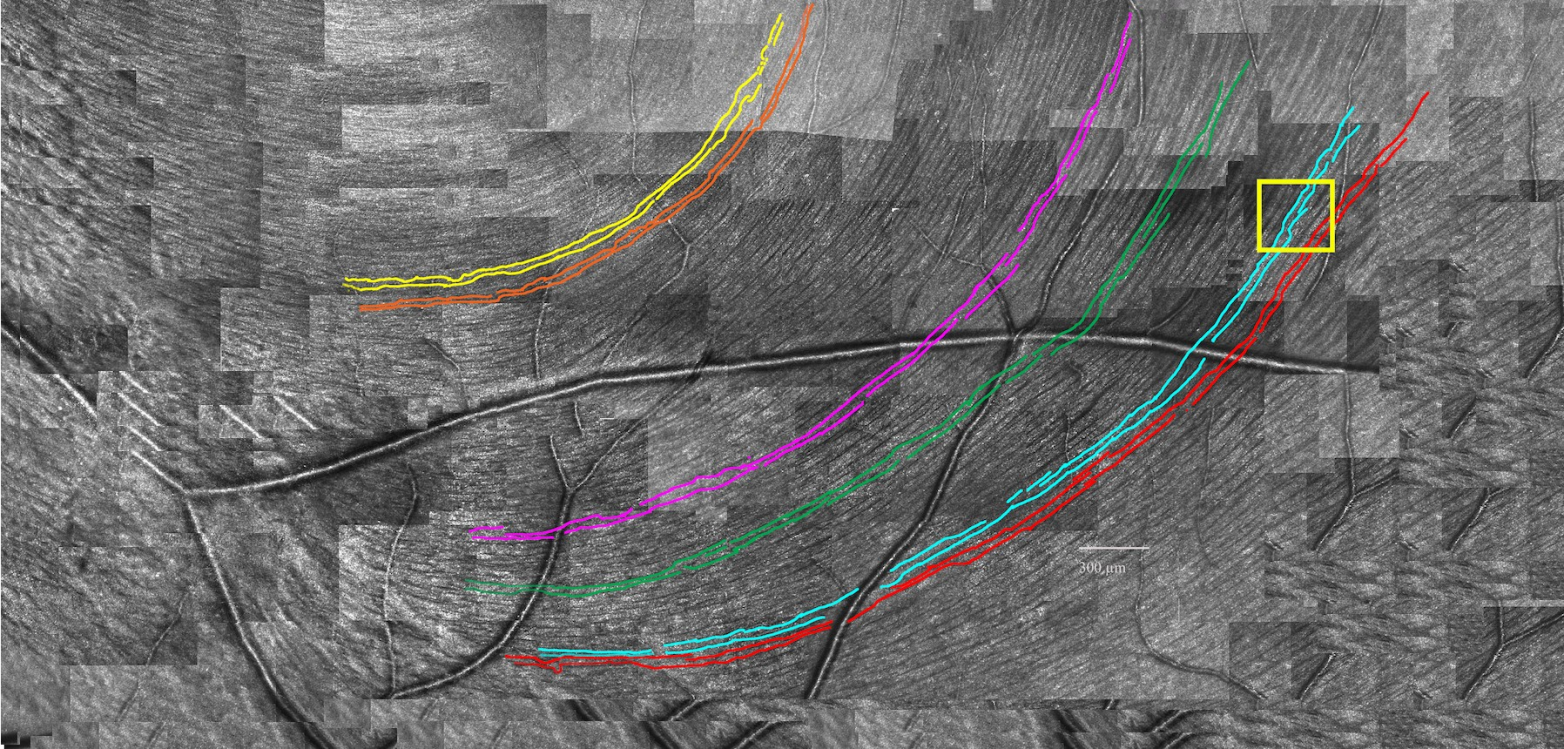
Bottom half of montage of AOSLO images of RNFL used in the pilot study. Colored curves show the six manually traced RNFBs. Yellow rectangle shows region that is presented at higher magnification in Fig 4.

The first step was to measure RNFB widths in a single large retinal montage, parts of which are shown in Huang et al. [5] As shown in Fig 1, selected RNFBs in inferior retina were traced at 4°, 8° and 10° from the fovea for inferior retina. RNFBs were selected that were continuous from the bottom of the fovea to near the temporal raphe. The montage did not have as much area for superior retina as inferior retina, so three regions were selected in superior retina in locations corresponding to the three inferior regions for which there was corresponding superior retina.

A total of 6 RNFBs were traced in inferior retina, in 3 groups of 2 each. For each of the 3 groups, widths were measured at the left and right ends of each pair of bundles; widths were measured for all RNFBs between the 2 RNFBs that were traced, and all RNFBs within the rectangles placed in superior retina. The number of RNFB widths that were measured ranged from 7 to 11 in given locations, yielding a total of 79 width measurements. To assess consistency of the staff member’s manual measurements, the 79 RNFB widths were measured twice, in two separate sessions masked from each other. The results of this pilot indicated considerable variability (see Results) and formed the basis of the method for the main study.

### Main study: Choosing regions of interest

**Fig 2:**
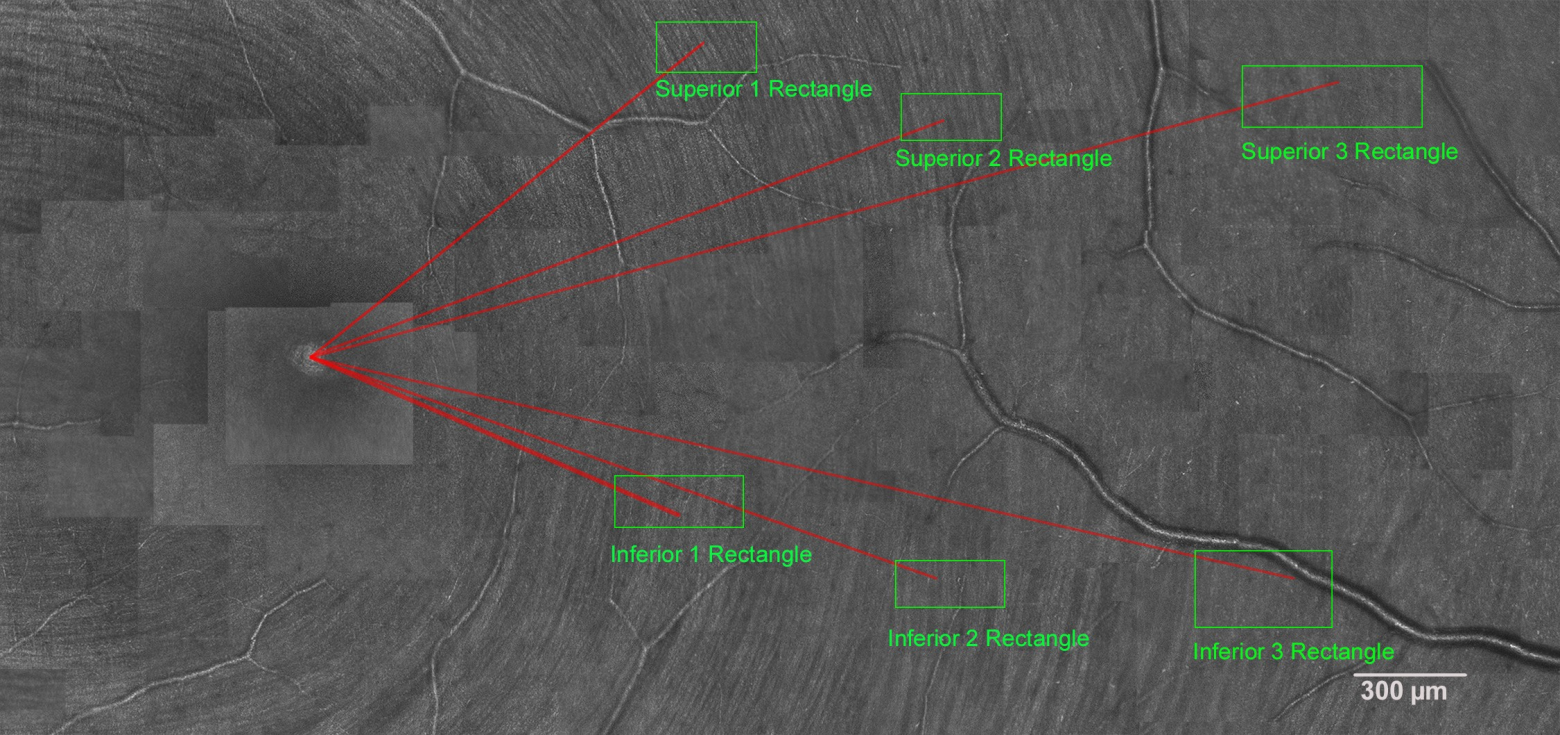
Example montage of AOSLO images of RNFL in the main study, showing the six retinal regions where RNFB widths were measured (green boxes). Thin red lines project from the center of the fovea to the regions where RNFB widths were measured; angles and lengths are given in Table 1.

For the main study we chose six regions of interest, 3 each in superior and inferior temporal retina. Fig 2 shows the positions of the rectangles from which the RNFB widths were determined. These locations were selected for each eye by drawing lines from the center of the fovea at the distances and angles specified in Table 1.

**Table 1:** Locations of the six retinal regions where RNFB width was measured in the 10 young adults free of eye disease.

Area	Distance of line from the Fovea (Degrees of visual angle)	Angle of the line from the Fovea (Degrees of visual angle)
Superior 1	4.6	39
Superior 2	6.2	20
Superior 3	9.8	18
Inferior 1	3.7	-23
Inferior 2	6.1	-19
Inferior 3	9.2	-13

**Fig 3:**
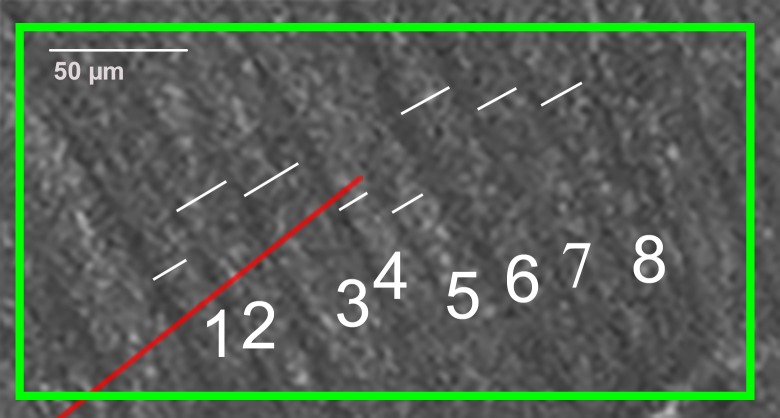
Region “Superior 1” from Fig 2, showing the 8 RNFBs and the RNFB measurement lines used to measure their widths. Red line is the tip of the line from the center of the fovea.

Fig 3 shows a close-up of a region of interest (Superior 1) where each individual RNFB measurement line can be seen. The end point of each line from the fovea determined the location of the 8 adjoining RNFBs of interest, and the nearest RNFB was considered the 4th bundle. When perpendicular, RNFBs 1 to 3 were the left of the 4th RNFB and RNFBs 5 to 8 were to the right of the 4th bundle. For each bundle, one RNFB measurement line was drawn that measured the width of the bundle. The actual position of the RNFB measurement line was determined by the following factors: clarity of the bundle, proximity of other RNFB measurement lines, proximity to the end point of the line from the fovea, and the shape of the RNFB along that particular section.

### Data sharing

The raw data and summary statistics are given in spreadsheets in the Supporting Information. In compliance with NIH and Indiana University policies and to protect the confidentiality of our human subject data and protected health information (PHI), Indiana University School of Optometry shares research images in the form of a limited data set pursuant to an approved data use agreement. AOSLO images used in this project will be shared with any research team whose institution executes an approved data use agreement with Indiana University.

## Results

**Fig 4:**
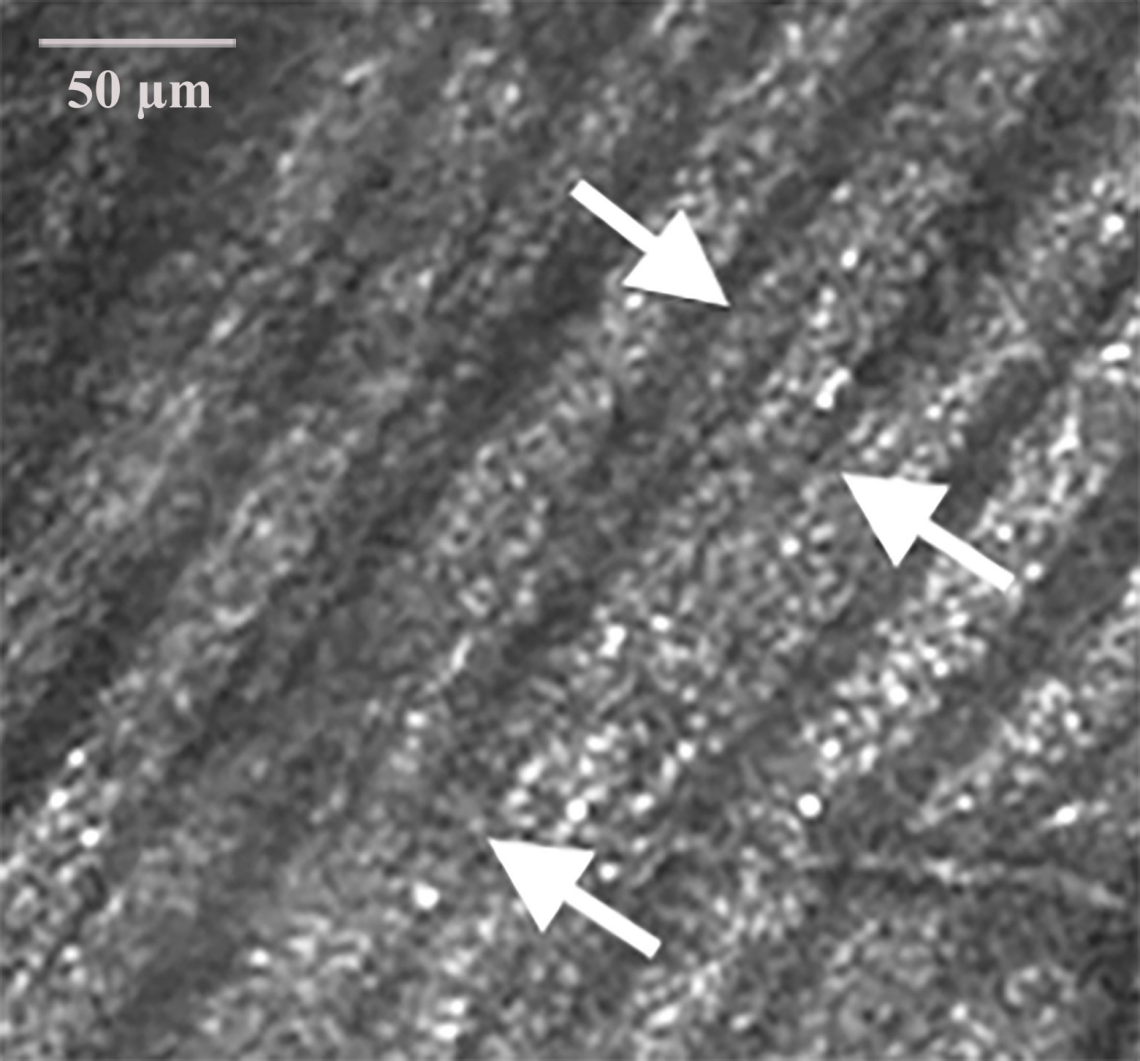
Close-up of a region of RNFL where individual RNFBs split and merge, indicated by the arrows. This is the region outlined with a yellow rectangle in Fig 1.

In the pilot study, we found that agreement between the repeated bundle width measurements was usually good: the mean difference between the two measurements (second width minus first width) was -1 μm, and the interquartile interval for differences ranged from -3 μm to +1 μm. However the full range was -14 μm to +9 μm, and 15% of the measured differences had absolute values greater than 5 μm. Examination of these cases found that the variability was due to small changes in locations of the RNFB width measurement between measurement sessions, in areas where RNFBs were merging together or branching apart. An example of such a region is shown in Fig 4.

**Fig 5:**
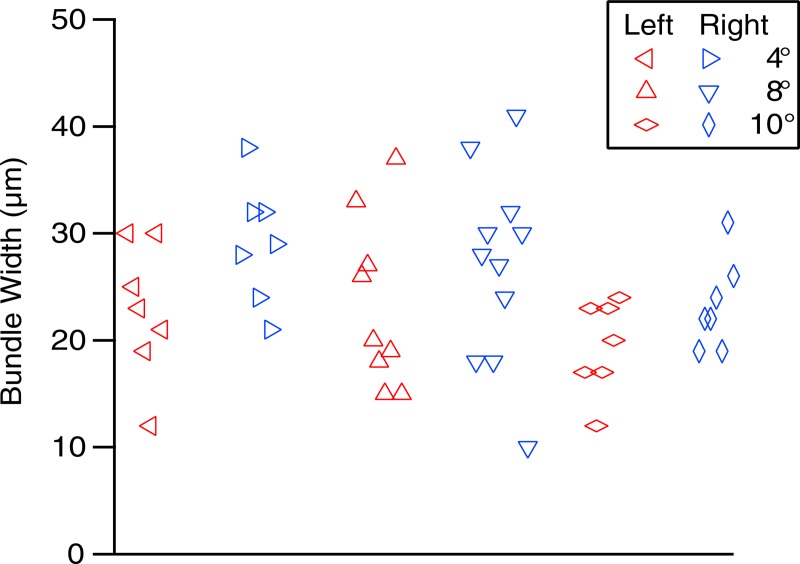
RNFB widths for the pilot study, using the montage in Fig 1, for the left and right ends of the three pairs of bundles.

Fig 5 shows individual RNFB widths measured in the pilot study. Individual RNFB widths ranged from 10 μm to 44 μm (median 22 μm), with considerable variability for each location and the range overlapping for all 6 locations. The mean bundle width was 22 μm for the bundles on the left and 24 μm for the bundles on the right. From the pilot study we concluded that the staff member had good consistency in the measurements, but that there was biological variability in the widths.

**Fig 6:**
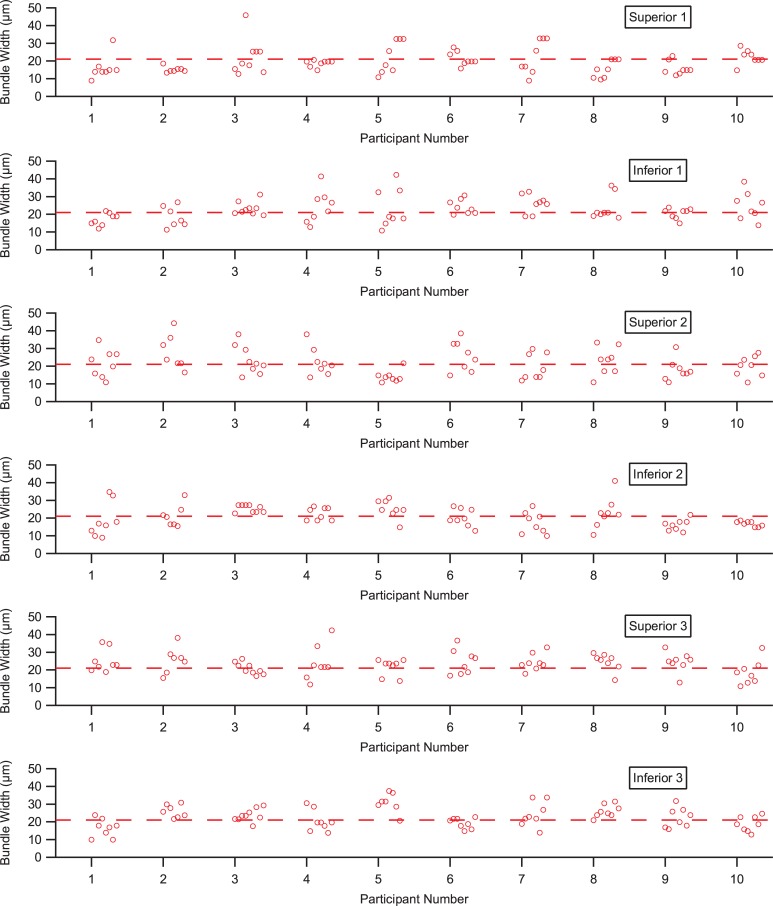
Widths of the 480 RNFBs measured in the group of 10 young adults free of eye disease, grouped by the six regions shown in Fig 2. Dashed line shows 21 μm, the median bundle width for the 480 RNFBs.

**Fig 7:**
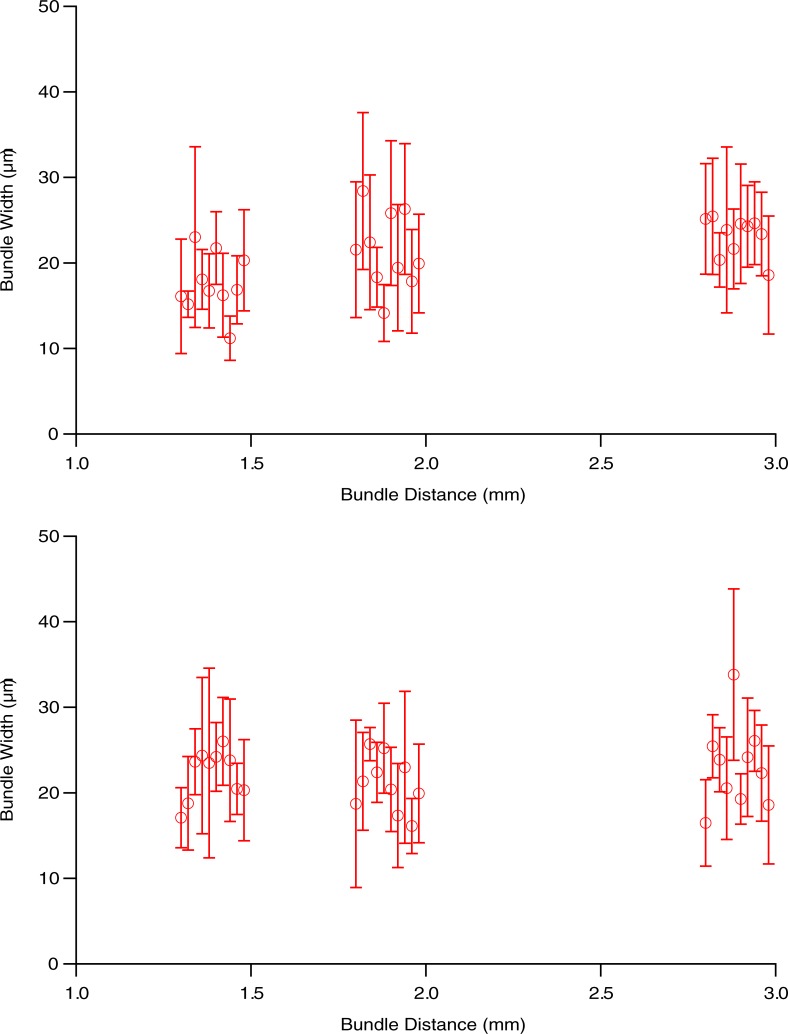
Means RNFB widths for the six regions identified in Fig 2, upper plot shows superior locations, lower plot shows inferior locations. Each mean is for one eye. Error bars show ± 1 standard deviation of the mean. Datapoints are displaced horizontally for clarity, in fact the same bundle distances were used in all 10 eyes.

Fig 6 shows all 480 RNFB widths for the 10 people in the main study. RNFB widths ranged from 9 μm to 55 μm, with a median of 21 μm and an interquartile interval from 16 μm to 26 μm. Every eye had large within-subject variability (range of 20–34 μm) in RNFB width for nearby bundles. Fig 7 shows means and standard deviations for each subject at each location. There was considerable overlap across individuals and locations.

Axial lengths for these 10 eyes ranged from 23.4 mm to 25.3 mm, with a median of 24.2 mm. For correlation of RNFB width with axial length, Pearson’s r was -0.07 for corrected widths and +0.40 for uncorrected widths.

## Discussion

We confirmed and extended the finding from the Miller lab [4] that there is large within-subject variability in width of individual RNFBs, which implies that the method for choosing which bundles to measure is a source of potential bias in comparing bundle widths both over time and between subject groups. We confirmed the finding from the Takayama lab [6] that between-subject variability of mean RNFB width had standard deviations of 3 μm to 6 μm; however, this suggests only that while the central tendency can be measured, comparisons between subjects would require handling the very large within-subject variability. We found that manual measurements were usually highly repeatable, within ±2 μm except when portions of an RNFB split off and joined another RNFB (Fig 4). The fact that AOSLO imaging shows RNFL bundles can combine and split apart has been noted before [5] and is consistent with histological studies. [28] This means that RNFB width varies considerably depending on the precise choice of where measurements of even an individual bundle is made, again providing a source of bias.

Because there was a report suggesting that glaucoma impacted RNFB width near the optic disc, [23] our original goal was to determine whether reflectance defects in en face SD-OCT images were due to decreased RNFB width or decreased RNFB reflectance. However, the wide variability in RNFB widths, even within a given region of retina, together with the variation in width of even a single RNFB with small changes in location of measurement, places constraints on the use of width measurements since results could be susceptible to selection bias.

The Takayama lab [6] did not discuss within-subject variability in RNFB width, but between-subject variability in RNFB width can be inferred from their means and standard deviations. For each eye, they had two graders each measure 3 widths per RNFB, and took the mean as the width of an individual RNFB. They then averaged widths across ~12 RNFBs per region, which should reduce within-subject variability. From their data, we calculated that between-subject coefficients of variation ranged from 0.1 to 0.3 across different regions, with a median of 0.2. From our data with one width measurement per RNFB, we found that within-subject variability had coefficients of variation from 0.1 to 0.5, with a median 0.3. A sample size of 8 RNFBs will tend to underestimate variance, so these are likely to be underestimates of within-subject variability. The large within-subject variability in RNFB width, and the branching and joining of RNFBs, leads us to conclude that RNFB width measurements are not likely to be a sensitive and reliable measure for evaluating glaucoma status in individual patients. It will be influenced by bias in selecting where to measure RNFB widths. Thus, while width measurements may be useful in longitudinal studies of individual patients if precise locations can be repeatably compared, or for evaluating population properties, applying them to evaluation of the health of individual patients will be severely constrained.

## Supporting information

S1 Table Raw data shown in Figs 5–7. The worksheet labeled"Fig 6" contains the raw data, which are plotted in Fig 6.
The worksheets labeled "Fig 5" and "Fig 7" show summary data plotted in those figures.
Worksheet “Fig 5”: Data in each column show RNFB widths for 1 young adult free of eye disease, for left and right values of eccentricity 4, 8, and 10 percent.
Worksheet “Fig 6”: Data in columns C-H show the RNFB widths of all 480 locations measured in 10 additional young adults free of eye disease. Column names indicate the region each bundle was located.
Worksheet “Fig 7”: Data in columns C and D show the mean and SD data plotted in Fig 7 for superior and inferior locations of each subject.
RNFB = retinal nerve fiber bundle
SD = standard deviation.
(XLSX)
