# Correction: Within-subject variability in human retinal nerve fiber bundle width

**DOI:** 10.1371/journal.pone.0229865

**Published:** 2020-02-27

**Authors:** The PLOS ONE Staff

There are a number of errors in the image for Fig 1 “Bottom half of montage of AOSLO images of RNFL used in the pilot study.” Please see the complete, correct Fig 1 here. The publisher apologizes for the error.

**Fig 1:**
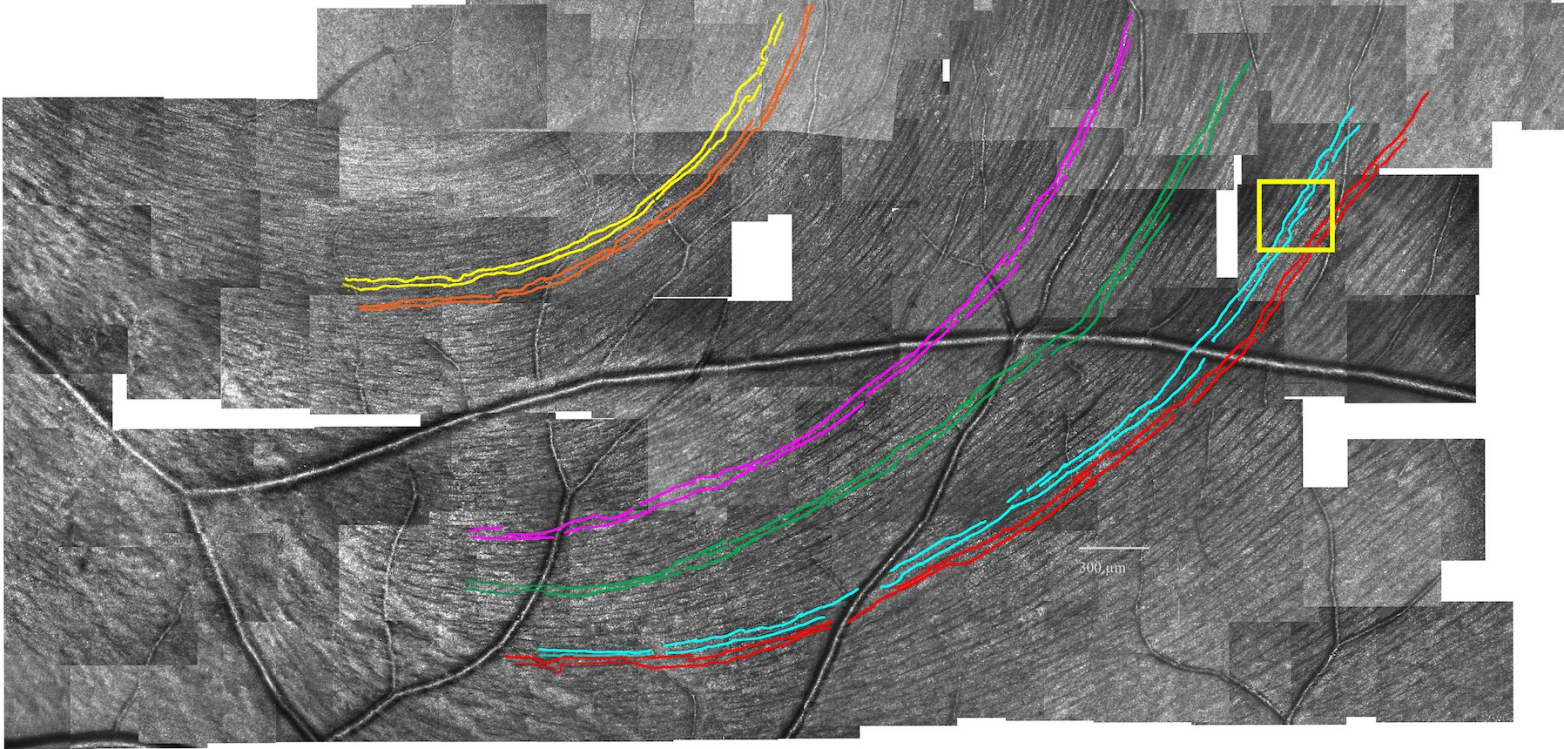
Bottom half of montage of AOSLO images of RNFL used in the pilot study. Colored curves show the six manually traced RNFBs. Yellow rectangle shows region that is presented at higher magnification in Fig 4.
